# Correction: The impact of factors on information sharing: An application of meta-analysis

**DOI:** 10.1371/journal.pone.0296555

**Published:** 2023-12-29

**Authors:** Chau Thi Diem Le, Miklós Pakurár, István András Kun, Judit Oláh

There are errors in the Funding section. The correct Funding statement is: This paper is supported by EFOP-3.6.3—VEKOP-16-2017-00007- "Young researchers for talent"—supporting careers in research activities in higher education program.
